# Room-Temperature All-solid-state Rechargeable Sodium-ion Batteries with a Cl-doped Na_3_PS_4_ Superionic Conductor

**DOI:** 10.1038/srep33733

**Published:** 2016-09-20

**Authors:** Iek-Heng Chu, Christopher S. Kompella, Han Nguyen, Zhuoying Zhu, Sunny Hy, Zhi Deng, Ying Shirley Meng, Shyue Ping Ong

**Affiliations:** 1 Department of NanoEngineering, University of California, San Diego, 9500 Gilman Drive, Mail Code 0448, La Jolla, CA 92093, USA

## Abstract

All-solid-state sodium-ion batteries are promising candidates for large-scale energy storage applications. The key enabler for an all-solid-state architecture is a sodium solid electrolyte that exhibits high Na^+^ conductivity at ambient temperatures, as well as excellent phase and electrochemical stability. In this work, we present a first-principles-guided discovery and synthesis of a novel Cl-doped tetragonal Na_3_PS_4_ (t-Na_3−*x*_PS_4−*x*_Cl_*x*_) solid electrolyte with a room-temperature Na^+^ conductivity exceeding 1 mS cm^−1^. We demonstrate that an all-solid-state TiS_2_/t-Na_3−*x*_PS_4−*x*_Cl_*x*_/Na cell utilizing this solid electrolyte can be cycled at room-temperature at a rate of C/10 with a capacity of about 80 mAh g^−1^ over 10 cycles. We provide evidence from density functional theory calculations that this excellent electrochemical performance is not only due to the high Na^+^ conductivity of the solid electrolyte, but also due to the effect that “salting” Na_3_PS_4_ has on the formation of an electronically insulating, ionically conducting solid electrolyte interphase.

Rechargeable all-solid-state sodium-ion batteries (ss-SIBs), which utilize ubiquitous sodium sources, are a promising low-cost, high-safety alternative to today’s lithium-ion batteries, especially for large-scale energy storage applications^1^,^2^,^3^,^4^,^5^. However, a critical challenge in the development of ss-SIBs is the lack of a sodium solid electrolyte with high ionic conductivity at ambient temperatures and a wide electrochemical window. Although oxide solid electrolytes such as *β*-alumina and NAtrium (or sodium) SuperIonic CONductors (NASICON) are well known, they exhibit reasonable ionic conductivities only at higher temperatures^1^,^6^,^7^,^8^,^9^,^10^. Moreover, their synthesis requires high-temperature processing to reduce the grain-boundary resistance, which is incompatible with traditional cathode materials and thus requires special fabrication procedures^1^.

Chalcogenide-based (S, Se) chemistries offer the potential for higher ionic conductivities than oxides^11^,^12^,^13^,^14^,^15^,^16^,^17^,^18^,^19^,^20^. Though it is likely that sulfide and selenide-based solid electrolytes may exhibit lower *intrinsic* electrochemical stability, the formation of passivating phases at the electrode-solid electrolyte interface can potentially mitigate further reactions^21^,^22^,^23^. Sulfide electrolytes, particularly sodium sulfides, also tend to be softer than oxides^24^, which allows intimate contact between electrode and solid electrolyte to be achieved via cold pressing instead of high-temperature sintering.

In recent years, several sodium superionic conductors with ionic conductivities approaching that of traditional organic electrolytes have been identified. For example, the cubic phase of Na_3_PS_4_ (c-Na_3_PS_4_) was first reported by Hayashi and co-workers in 2012 with a measured Na^+^ conductivity of 0.2 mS cm^−1^ 25. The crystal structure of c-Na_3_PS_4_ has the 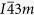 space group and is the high-temperature^26^, disordered polymorph of tetragonal Na_3_PS_4_ (t-Na_3_PS_4_) with space group 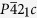
27. Since the discovery of c-Na_3_PS_4_, there have been a number of successful efforts at further enhancing the room temperature conductivity of Na_3_PS_4_ systems^26^,^28^,^29^, with the highest value thus far of 0.74 mS cm^−1^ achieved within the (1-*x*)c-Na_3_PS_4_-*x*Na_4_SiS_4_ pseudo-binary system at *x* around 0.06^29^. A recent first principles investigation by some of the authors of this work proposed that Na-excess-induced Na disorder is the reason for the high conductivity observed in c-Na_3_PS_4_, and that Sn^4+^ cation doping (for P^5+^) may yield greater improvement in conductivity than Si^4+^ doping but at the expense of higher dopant formation energy^30^.

The introduction of defects via aliovalent doping is a common strategy to improve the ionic conductivity of materials. For Na_3_PS_4_, previous efforts have mainly focused on the cubic phase and the introduction of Na excess interstitials via substitution of P^5+^. An alternative strategy of aliovalent doping is to introduce Na^+^ vacancies in Na_3_PS_4_. Halide (X^−^) anion doping (for S^2−^) is a potential strategy for Na^+^ vacancy creation^31^. However, previous experimental efforts with (1-*x*)c-Na_3_PS_4_-*x*NaI glass-ceramics achieved a Na^+^ conductivity of ~0.1 mS cm^−1^ 32, which is lower than that of the undoped and Si-doped c-Na_3_PS_4_. Furthermore, an unknown phase was found as the major precipitant when NaI was introduced at *x* ≥ 0.1, suggesting an intrinsic incompatibility of I^−^ ions with the S^2−^ host at such high doping levels.

In this work, we demonstrate the stable cycling of a TiS_2_/t-Na_3−*x*_PS_4−*x*_Cl_*x*_/Na ss-SIB at room temperature, with a capacity of about 80 mAh g^−1^ at a rate of C/10 over 10 cycles for the first time. The key enabler to this outstanding cell performance is a novel Cl-doped t-Na_3_PS_4_ solid electrolyte with formula Na_2.9375_PS_3.9375_Cl_0.0625_, which was developed using a tightly integrated combination of density functional theory (DFT) calculations, synthesis and characterization. From DFT calculations, we show that Cl^−^ exhibits good chemical compatibility with the S^2−^ host, with low dopant formation energy and similar ionic radius. The concomitant introduction of Na vacancies results in a predicted room-temperature Na^+^ conductivity exceeding 1 mS cm^−1^. These predictions are confirmed through experimental synthesis of tetragonal Na_2.9375_PS_3.9375_Cl_0.0625_, and its demonstration in a ss-SIB architecture. We will also present evidence from DFT computations that suggest that the superior performance achieved in this cell is not only the result of the high Na^+^ conductivity of the solid electrolyte, but also the formation of electronically-insulating, ionically-conducting passivation layers at the electrode-solid electrolyte interface due to the presence of the Cl^−^ anion.

## Results

Using DFT calculations, we first performed a systematic investigation of the dopability of potential halide X^−^ (X = F, Cl, Br and I) into both the tetragonal and cubic phases of Na_3_PS_4_. As the DFT predictions for both phases are extremely similar, we will henceforth present only the results of the more stable tetragonal phase for brevity, and interested readers are referred to the Supplemental Information for the results on the cubic phase.

### Dopability of Na_3_PS_4_

**Figure 1:**
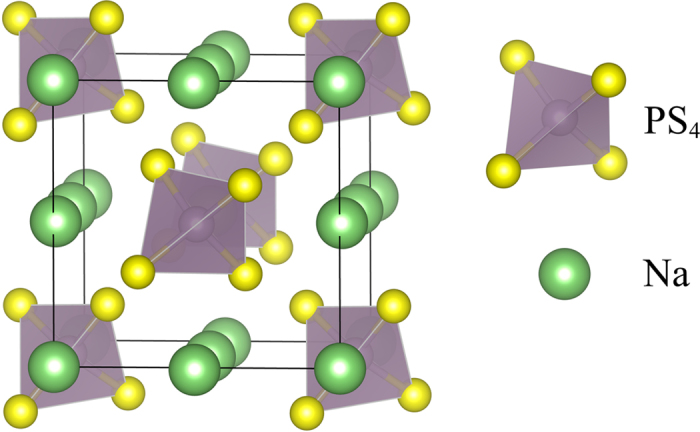
Crystal structure of pristine t-Na_3_PS_4_. The tetragonal polymorph of the Na_3_PS_4_ crystal. There are symmetrically distinct Na sites in t-Na_3_PS_4_, Na1 (4*d*) and Na2 (2*a*), and the PS_4_ tetrahedra are centered at the 2*b* positions.

Figure 1 shows the crystal structure of t-Na_3_PS_4_. There are two symmetrically distinct Na sites in t-Na_3_PS_4_, Na1 (4*d*) and Na2 (2*a*), and the PS_4_ tetrahedra are centered at the 2*b* positions^27^. The symmetrically distinct Na sites in c-Na_3_PS_4_ are labeled similarly as cubic Na1 (6*b*) and Na2 (12*d*). The Na1 (4*d*) and Na2 (2*a*) sites in t-Na_3_PS_4_ essentially occupy the same positions as the Na1 (6*b*) sites in c-Na_3_PS_4_^31^.

A single halide dopant was introduced into a 2 × 2 × 2 supercell of t-Na_3_PS_4_ by replacing one of the S atoms with X, and simultaneously a Na vacancy was introduced to form Na_47_P_16_S_63_X, or t-Na_3−*x*_PS_4−*x*_X_*x*_ with *x* = 0.0625. All symmetrically distinct 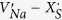 configurations (Kröger–Vink notation is adopted) were evaluated, and the lowest energy configuration was used for subsequent analyses.

Table 1 summarizes the halide dopant formation energies. We find that all halides (F, Cl, Br and I) are predicted to have relatively low formation energies at a doping level of *x* = 0.0625. Fluoride doping is predicted to be the most favorable (*E*_*f*_ = 0.76 eV/F^−^), followed by chloride (*E*_*f*_ = 0.96 eV/Cl^−^) and iodide (*E*_*f*_ = 0.99 eV/I^−^). Bromide doping is by far the least favorable with *E*_*f*_ = 1.11 eV/Br^−^. Higher doping levels result in a significantly higher dopant formation energy, e.g., *E*_*f*_ = 1.77 eV when doping Cl^−^ in the same supercell size at *x* = 0.125. For all doped structures, the lowest energy structure has the X^−^ anion substituted in the S^2−^ (8*e*) site with a vacancy on the Na2 site. Nevertheless, the energy differences between different 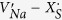 orderings are generally small (<10 meV/atom), which suggest that the dopants and vacancies are likely to be disordered at room temperature.

Despite the fact that F^−^ doping is predicted to be the most favorable, we have selected Cl^−^ doping for further investigation due to several considerations. First, NaCl, aka table salt, is by far a more commonly available precursor than NaF. Second, fluoride chemistry inherently comes the possibility of HF exposure. Finally, the Cl^−^ anion has an ionic radius that is closest to S^2−^, which would minimize the local structural distortion introduced at the substituted site. The doping concentration of Cl is fixed at *x* = 0.0625, given that previous work in doping I^−^ (which has a similar dopant formation energy at the same concentration) into c-Na_3_PS_4_ at *x* > 0.1 resulted in formation of unknown phases^32^.

### Ionic conductivity of Cl-doped t-Na_3_PS_4_

As demonstrated in recent work by some of the co-authors^30^, pristine c-Na_3_PS_4_, i.e., without interstitial or vacancy defects, is predicted to be an extremely poor ionic conductor in AIMD simulations. Using AIMD simulations (see Methods section), a similar result is obtained with t-Na_3_PS_4_ in this work, which is not surprising given that the small differences in lattice parameters and atomic positions between the cubic and tetragonal polymorphs.

**Figure 2:**
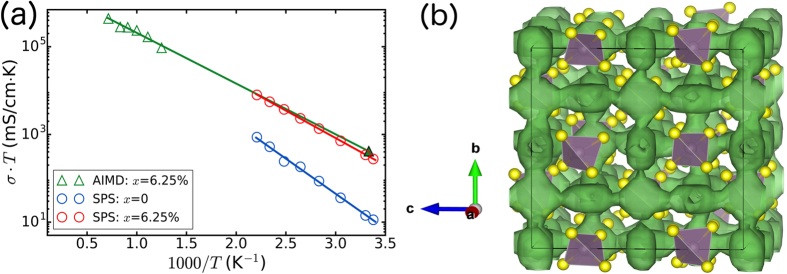
Ionic conductivity and Li^+^ probability density distribution of t-Na_3−*x*_PS_4−*x*_Cl_*x*_. (**a**) Arrhenius plots of the t-Na_3−*x*_PS_4−*x*_Cl_*x*_ with *x* = 0 (blue) and 6.25% (red) obtained from SPS measurements, and *x* = 6.25% (green) from AIMD simulations. The filled green triangle indicates the extrapolated ionic conductivity at 300 K from AIMD simulations. (**b**) Isosurface of the Na^+^ probability density distribution (*P*, in green) in the t-Na_3−*x*_PS_4−*x*_Cl_*x*_ (*x* = 6.25%) at 800 K, with *P* = 0.0001 *a*_0_^−3^ (*a*_0_ is the Bohr radius).

Figure 2a shows the Arrhenius plot of the log of the conductivity-temperature product (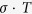) versus 1/*T* for t-Na_2.9375_PS_3.9375_Cl_0.0625_ obtained from AIMD simulations. With the introduction of a 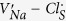 defect pair, the Na^+^ conductivity at 300 K is predicted to be 1.38 mS cm^−1^ with an activation barrier of 232 meV (see Table 2). During the preparation of this article, it has come to our attention that Klerk *et al*. has also performed AIMD simulations on halide doping in t- and c-Na_3_PS_4_^31^. Though the qualitative conclusions of vacancy-induced conductivity are similar, we note that Klerk *et al*. only performed relatively short AIMD simulations at a single temperature of 525 K; room-temperature Na^+^ conductivities and activation energies were therefore not obtained.

From the Na^+^ probability density distribution (Fig. 2b), we may observe that t-Na_2.9375_PS_3.9375_Cl_0.0625_ is predicted to be a 3D diffuser comprising of chains of Na1 sites along the c direction interconnected via the Na2 sites, which form a body-centered cubic sublattice. Such a 3D diffusion network is expected to be highly robust against the potential introduction of blocking defects^33^.

### Electrochemical stability of t-Na_3−*x*_PS_4−*x*_Cl_*x*_

Besides high ionic conductivity, an effective solid electrolyte candidate should also exhibit good electrochemical stability against the electrodes. Electrochemical stability may be achieved in two ways. First, the solid electrolyte can be intrinsically inert against any reaction with the electrodes. However, due to the high reactivity of Na metal and the highly oxidizing nature of the most charged high-voltage cathodes, it is difficult to find a material that is intrinsically stable over such a wide range of sodium chemical potential^17^,^23^. A second, more achievable option is to optimize the electrode-solid electrolyte chemistry as a whole such that good passivation layers are formed at the electrode-solid electrolyte interfaces that act as a barrier against further reaction. A good passivation layer should have a reasonable Na^+^ conductivity, and low electronic conductivity.

**Figure 3:**
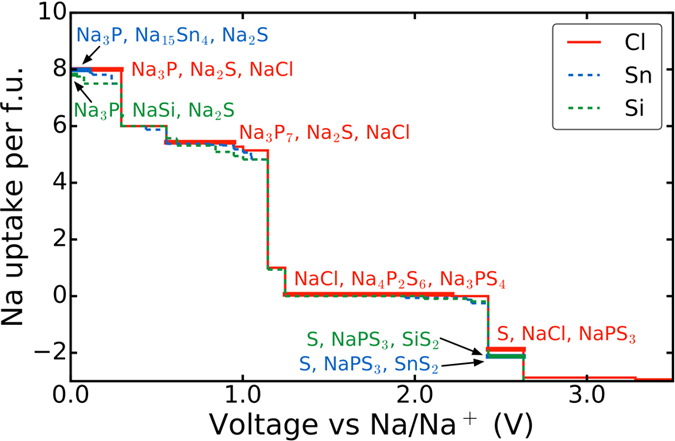
Electrochemical decomposition products of doped Na_3_PS_4_ compounds. Plots of Na uptake per formula unit (f.u.) of t-Na_2.9375_PS_3.9375_Cl_0.0625_ (red solid), c-Na_3.0625_Sn_0.0625_P_0.9375_S_4_ (blue dashed) and c-Na_3.0625_Si_0.0625_P_0.9375_S_4_ (green dashed) solid electrolytes against voltage vs Na/Na^+^. At low voltage (high Na chemical potential), each solid electrolyte undergoes reduction and uptakes Na, while at high voltage (low Na chemical potential), each solid electrolyte is oxidized and loses Na. Text indicates the predicted phase equilibria at corresponding regions of the profile. Only selected regions are annotated for brevity.

Figure 3 shows the Na grand potential phase stability plot of the t-Na_2.9375_PS_3.9375_Cl_0.0625_ solid electrolyte as a function of Na chemical potential. We find that at the Na metal anode (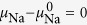 eV), the predicted phase equilibria comprises Na_2_S, NaCl and Na_3_P in the ratio of 63:1:16. The dominant phase Na_2_S is a good electronic insulator with PBE band gap of 2.4 eV, and NaCl has a PBE band gap of 5.0 eV^34^. Na_3_P has a small PBE band gap of 0.4 eV (the screened hybrid HSE functional^35^,^36^ gives a band gap of 0.76 eV), but is not expected to dominate the conductivity characteristics of the anode/electrolyte interface. The predicted phase equilibria are very similar to those of pristine t-Na_3_PS_4_ that consists of Na_2_S and Na_3_P in the ratio of 4:1. It should be noted that the PBE functional tends to severely underestimate band gaps, and the true band gaps are likely to be even higher. These phases are expected to exhibit moderate Na^+^ conductivity, particularly in an amorphous solid-electrolyte interphase (SEI) layer.

At voltages above 2.4 V versus Na/Na^+^, we find that the t-Na_2.9375_PS_3.9375_Cl_0.0625_ solid electrolyte is predicted to be unstable against Na extraction to form NaPS_3_ + S + NaCl. However, if the operating voltage is kept below 2.4 V, the predicted phase equilibria at the charged cathode-solid electrolyte interface retains t-Na_3_PS_4_ + NaCl as the primary component.

### Synthesis and characterization of t-Na_3−*x*_PS_4−*x*_Cl_*x*_

Pure t-Na_3_PS_4_ was synthesized from Na_2_S and P_2_S_5_ precursors. The Cl^−^ dopant was introduced by adding NaCl following the chemical reaction (1.5 − *x*)Na_2_S + 0.5P_2_S_5_ + *x*NaCl → Na_3−*x*_PS_4−*x*_Cl_*x*_. The resulting pellets were then densified via spark plasma sintering (SPS) to minimize porosity of the solid electrolyte. The synthesis details are given in the Methods section, and the dimensions and density of the pristine and doped pellets are provided in Supplementary Information (see Table S1).

**Figure 4:**
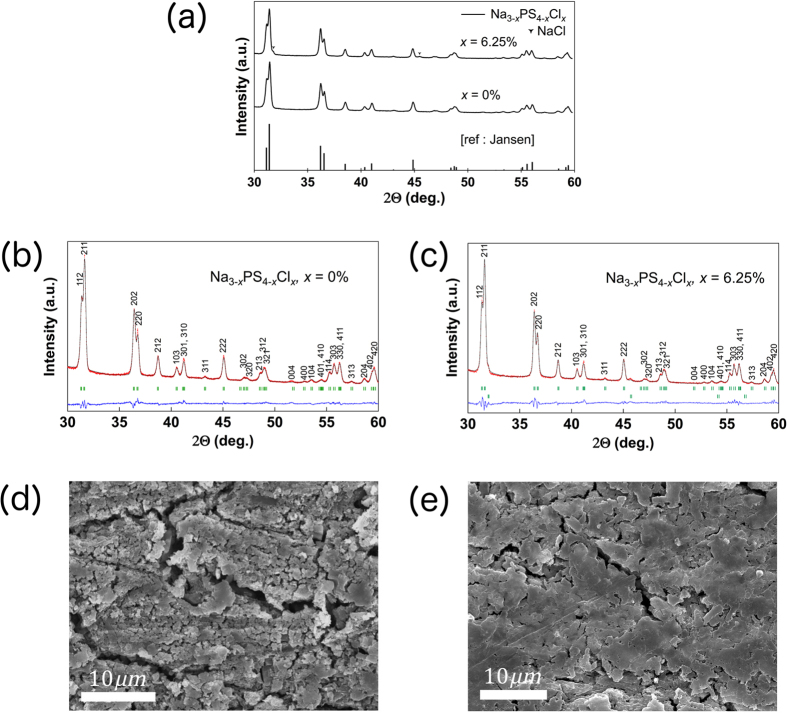
Characterization and morphology of t-Na_3−*x*_PS_4−*x*_Cl_*x*_. (**a**) XRD patterns for t-Na_3−*x*_PS_4−*x*_Cl_*x*_ with *x* = 0% and 6.25%, and previous study in ref. 27. (**b**) Refinement plot of the pristine t-Na_3_PS_4_. (**c**) Refinement plot of Cl-doped t-Na_3_PS_4_. Solid red and black lines denote the observed and calculated XRD patterns, while the green ticks mark the position of the reflections allowed by the space groups of t-Na_3_PS_4_ (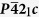) and NaCl (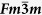). The difference between the observed and calculated patterns is signified by the blue line. (**d**) SEM image of pristine t-Na_3_PS_4_ SPS sample, and (**e**) SEM image of SPS sample of doped t-Na_3−*x*_PS_4−*x*_Cl_*x*_ (*x* = 6.25%). Scale bar is 10 *μ*m.

Figure 4a shows the XRD data for the two compositions, t-Na_3−*x*_PS_4−*x*_Cl_*x*_ with *x* = 0% and 6.25%. At *x* = 0%, we identify the crystalline phase formed to be t-Na_3_PS_4_, and the XRD pattern is in excellent agreement with the previous study by Jansen *et al*^27^. With the addition of chloride via NaCl at *x* = 6.25%, the tetragonal phase is retained with trace amounts of unreacted NaCl, and no reflections from unknown crystals are present in the spectra. Additionally, we observe a slight increase in the peak intensities of all the XRD reflections, with the most significant change occurring in the high index peaks, (112) and (211), at about 31°. This observation is the first indication that aliovalent substitution of S^2−^ by Cl^−^ was successful, because the halogen has a higher scattering factor than sulfur.

Rietveld refinement calculations were first conducted for t-Na_3−*x*_PS_4−*x*_Cl_*x*_. To obtain a baseline of the crystal parameters, a refinement calculation was performed on the pristine (*x* = 0%) structure. The refined XRD pattern of the pristine structure is shown in Fig. 4b. The refined lattice constants for the pristine structure are in excellent agreement with the previously reported values^27^. These parameters were then used as an initial model to study the aliovalent substitution of S^2−^ by Cl^−^. Figure 4c shows the refined pattern of doped t-Na_2.9375_PS_3.9375_Cl_0.0625_. No unknown phase was detected in the crystal, and no side reactions were observed during the synthesis. Although a trace amount of NaCl was detected in the spectrum, our refinement results show that it comprises less than 1 at%.

Table 3 summarizes the crystallographic parameters (lattice constants, thermal factors, and atomic occupancies) of the pristine and doped solid electrolyte from the Rietveld refinement and DFT calculations, which are in excellent agreement. From the refinement calculations, we estimated an increase in the lattice volume associated with the substitution of S^2−^ by Cl^−^. This volumetric change is very small and also agrees well with the DFT values. With the introduction of Cl^−^, there is a corresponding decrease in sodium and sulfur occupancies and increase in their respective isotropic thermal factors (*B*_iso_). These observations can be attributed to the formation of the Na^+^ vacancy. In summary, the Rietveld refinement supports the successful incorporation of Cl^−^ into the S^2−^ sublattice, with the concomitant introduction of Na^+^ vacancies. More advanced characterization techniques such as solid-state nuclear magnetic resonance (NMR) may be employed to confirm the success of the doping in future work.

Cross-sectional SEM images of the pristine t-Na_3_PS_4_ and doped t-Na_2.9375_PS_3.9375_Cl_0.0625_ SPS samples under identical processing conditions are shown in Fig. 4d,e, respectively. We note that the Cl^−^ doping does not lead to any significant morphology changes. The images show that the local morphology of the pellets is densely formed due to SPS processing in both compounds. An EDX measurement was collected from the doped sample (see Figure S1 in Supplementary Information). From the EDX measurement, we determined that sulfur and chlorine are uniformly distributed throughout the sample, with no noticeable aggregation of element-rich domains. In conjunction with the refinement results, the majority of the chloride dopant is found to be integrated into the host crystal lattice.

### Conductivity measurements of t-Na_3−*x*_PS_4−*x*_Cl_*x*_

The experimental measurement of pristine t-Na_3_PS_4_ shows a low ionic conductivity of 0.05 mS cm^−1^ at 303 K, with an activation energy value of 317 meV (see Fig. 2a and Table 2). The t-Na_2.9375_PS_3.9375_Cl_0.0625_ solid electrolyte, on the other hand, shows an extremely high conductivity of 1.14 mS cm^−1^ at 303 K and a low activation barrier of 249 meV. The measured conductivity and activation barrier are in excellent agreement with the calculated values (see Table 2). The room temperature Nyquist plots for the pristine t-Na_3_PS_4_ and doped t-Na_2.9375_PS_3.9375_Cl_0.0625_ are given in Figure S2, where the total impedance in each structure was used to calculate the room temperature conductivities of each material. A significantly larger semi-circle is observed for the pristine t-Na_3_PS_4_ compared to doped t-Na_2.9375_PS_3.9375_Cl_0.0625_, indicating a much larger total resistance in the pristine t-Na_3_PS_4_.

### Electrochemical performance

**Figure 5:**
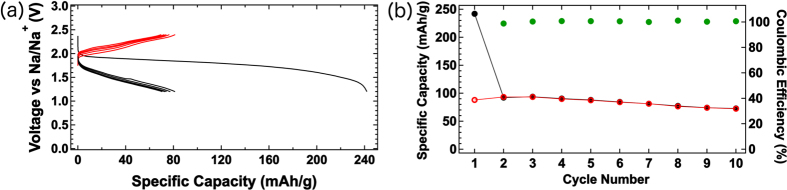
Electrochemical performance of TiS_2_/t-Na_2.9375_PS_3.9375_Cl_0.0625_/Na all-solid-state full cell. Charge-discharge profile of TiS_2_/t-Na_2.9375_PS_3.9375_Cl_0.0625_/Na full cell at room-temperature. Cell was cycled under constant current conditions with a current density of 0.149 mA cm^−2^ (C/10 rate) from 1.2 V to 2.4 V. The cell was able to routinely deliver 80 mAh g^−1^ over 10 cycles. Red and black lines in the charge-discharge profile denote charging and discharging, respectively. Similarly, red and black markers signify charge and discharge capacities, while the green circles mark the coulombic efficiency by cycle.

A full cell was constructed using a TiS_2_ charged cathode and a Na metallic anode. The choice of the TiS_2_ cathode is motivated by its suitable operating voltage (~1.7 V versus Na/Na^+^), which is well within the limits of the DFT predicted stability window of the t-Na_2.9375_PS_3.9375_Cl_0.0625_ solid electrolyte, as well as its fast kinetics for Na^+^ intercalation^37^. The cell was galvanostatically cycled from 1.2 V to 2.4 V. The cell was held for two minutes between switching from charging to discharging. A current density of 0.149 mA cm^−2^ was applied, corresponding to a C/10 rate. The theoretical capacity of the NaTiS_2_ active material is 198 mAh g^−1^. The discharge and charge capacity of the first cycle were ~240 mAh g^−1^ and 80 mAh g^−1^, respectively. The source of the excess capacity as well as the large irreversible capacity of the first cycle is currently under investigation, and will be the evaluated in a subsequent study of the interface stability and its effects on cyclability and longevity. Currently, impedance measurements during the first cycle clearly indicate the formation of stable interfacial phases at the solid-electrolyte/electrode interfaces (see Figure S3 in the Supplementary Information). The subsequent charge and discharge capacities of the cell over 10 cycles were ~80 mAh g^−1^, with a coulombic efficiency above 98% (see Fig. 5). Such a stable performance is consistent with the cyclic voltammetry results (see Figure S3 in the Supplementary Information) in which Cl-doped t-Na_3_PS_4_ is found electrochemically stable against the Na anode for up to 5 V. Strong polarization, common in Na-ion cells, was also observed at the point of switching from charging to discharging, and vice versa.

## Discussion

The design of an all-solid-state rechargeable battery is a multi-component, multi-property optimization effort; it is therefore insufficient to merely focus on bulk ionic conductivity of the solid electrolyte as the only target parameter. In this work, we have demonstrated how an integrated computational and experimental effort can significantly accelerate such multi-component, multi-property optimization, resulting in a promising new t-Na_2.9375_PS_3.9375_Cl_0.0625_ solid electrolyte that has been demonstrated in a full ss-SIB cell with good cyclability and capacity.

Aliovalent doping is a common strategy to introduce defects (vacancies or interstitials) into a solid electrolyte candidate to further enhance its conductivity. Surprisingly, we find halide doping in Na_3_PS_4_ to be somewhat more favorable than cation (Si^4+^, Ge^4+^ and Sn^4+^) doping, with slightly lower dopant formation energies of 0.76–1.11 eV vs 1.04–1.32 eV for cation^30^. Though dopant formation energies of 0.76–1.11 eV might appear at first glance to be relatively high, it should be noted that these values depend strongly on the chemical potential references used. At the elevated temperatures during synthesis, Na_2_S loss is likely to lower the chemical potentials of Na and S, significantly promoting 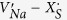
formation.

Both the cation + interstitials and anion + vacancies doping strategies are predicted to be effective in enhancing the ionic conductivity of Na_3_PS_4_. In fact, we find no evidence of any significant difference in the predicted bulk Na^+^ conductivities between the cubic and tetragonal Na_3_PS_4_, which is not surprising given the very small differences in lattice parameters between the two polymorphs^38^. We speculate that the nature of the defects present would promote the formation of one polymorph over another. Excess Na interstitials would need to occupy the cubic Na2 (12*d*) sites, promoting the formation of the disordered cubic phase, while Na vacancies with halide substitution would result in slight lattice expansion (due to reduced electrostatic attraction) and promote the formation of the tetragonal phase. Indeed, our attempts at synthesizing a phase-pure Cl-doped c-Na_3_PS_4_ phase has been unsuccessful, while the Cl-doped t-Na_3_PS_4_ was readily obtained.

The fundamental difference between cation M^4+^ and anion X^−^ doping is in the interfacial products that are predicted to form at the Na anode. For Si or Sn-doped Na_3_PS_4_, small-gap compounds such as Na_4_Si_4_ (space group of *C2*/*c*; PBE band gap of ~1.2 eV) and Na_15_Sn_4_ (space group of 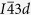; PBE band gap of ~0 eV) as well as Na_2_S and Na_3_P are predicted to form at the anode/solid electrolyte interface (see Fig. 3). For Cl-doped Na_3_PS_4_, on the other hand, the anode/solid electrolyte interface comprises predominantly Na_2_S with smaller amounts of NaCl and Na_3_P. Alkali halides are well-known components in the solid-electrolyte interphase (SEI) of rechargeable lithium-ion batteries, where LiF is formed from the reaction between the LiPF_6_ salt and the electrodes. Indeed, the “salting” of the SEI is likely to improve its ionic conductivity as Cl^−^ dopants will similarly be introduced to the amorphous Na_3_P + Na_2_S SEI. It should be noted that in all cases (undoped, cation-doped, and halide-doped), the small-gap compound Na_3_P (PBE band gap of ~0.4 eV) is also predicted to form at the Na anode, though it is not the dominant phase. Based on the achieved cycling performance in this work as well as in previous works on c-Na_3_PS_4_^25^, we would surmise that the effect of Na_3_P on the interfacial stability is small.

Ultimately, the total conductivity of a solid electrolyte depends not only on its bulk conductivity, but also the grain boundary contributions. In this respect, the specifics of the synthesis procedure are critically important. In this work, spark-plasma sintering was used to achieve a fully-dense solid electrolyte with reduced grain boundary resistance, and an overall conductivity exceeding 1 mS cm^−1^ that is very close to the DFT predicted bulk conductivity (see Fig. 2). This overall conductivity is higher than that of Si-doped c-Na_3_PS_4_ (0.74 mS cm^−1^)^29^ and is the highest value for sodium thiophosphates achieved thus far. Although the recently reported Na_3_PSe_4_ and Na_3_SbS_4_ have higher conductivities, the more expensive and less stable Se^2−^ anion is utilized in Na_3_PSe_4_^14^, while Na_3_SbS_4_ requires a more complicated electrolyte bilayer approach using c-Na_3_PS_4_ to stabilize the interface at the Na anode^20^.

We have demonstrated the potential of the t-Na_2.9375_PS_3.9375_Cl_0.0625_ solid electrolyte by integrating it in a ss-SIB full cell. In a full cell, the choice of the cathode and anode must be given careful consideration, as well as their interactions with the solid electrolyte. From the DFT grand potential analysis, we find that the t-Na_2.9375_PS_3.9375_Cl_0.0625_ electrolyte is predicted to be relatively stable up to ~2.4 V vs Na/Na^+^, while passivation is predicted to occur at the Na anode. Therefore, TiS_2_ was chosen as the cathode. At a current density of 0.149 mA cm^−2^, a cell capacity of ~80 mAh g^−1^ was achieved over 10 cycles of the TiS_2_/t-Na_2.9375_PS_3.9375_Cl_0.0625_/Na full cell. The increase in internal cell resistance, leading to capacity decay after subsequent cycling, is common when forming a SEI layer. Though the reversible capacity reported by Hayashi *et al*. for the c-Na_3_PS_4_ solid electrolyte is similar^25^, that performance was achieved with a much lower current density (0.013 mA cm^−2^) against a Na-Sn alloy as the anode. Though the Si-doped c-Na_3_PS_4_ and Na_3_PSe_4_ solid electrolytes have higher measured conductivities than c-Na_3_PS_4_, their room-temperature performance in a full ss-SIB cell has not yet been demonstrated^14^,^29^. To our knowledge, this is *the first time that cycling at a rate as high as C/10 has been demonstrated in a full ss-SIB with a Na metal anode at room temperature*.

## Conclusion

In conclusion, we have demonstrated the prediction and synthesis of a novel Cl-doped tetragonal Na_3_PS_4_ solid electrolyte, or t-Na_2.9375_PS_3.9375_Cl_0.0625_, and its good cycling performance in a full all-solid-state rechargeable sodium-ion cell at a rate of C/10. The predicted bulk and measured total conductivities of the t-Na_2.9375_PS_3.9375_Cl_0.0625_ solid electrolyte exceed 1 mS cm^−1^, which is one of the highest conductivity reported for any sodium superionic conductor thus far. More importantly, the “salting” of Na_3_PS_4_ is predicted to improve the characteristics of the interfacial phase equilibria at the anode/solid electrolyte interface, forming an electronically insulating and ionically conducting solid-electrolyte interphase. We also demonstrate the potential of spark-plasma sintering as a technique for achieving a dense sulfide electrolyte with reduced grain boundary resistance.

## Methods

### Density functional theory calculations

All DFT calculations were performed using similar methodologies as previous works by the authors^30^,^39^. For the sake of brevity, we refer interested readers to those works and only briefly summarize the key parameters here.

All calculations were performed with the Vienna Ab initio Simulation Package (VASP)^40^, within the projector augmented wave (PAW) approach^41^. The Python Materials Genomics (pymatgen) materials analysis library was used for all analyses^42^. All structural relaxations and total energy calculations were carried out using parameters similar to those used in the Materials Project^34^. The key parameters are the use of the Perdew-Burke-Ernzerhof (PBE) generalized-gradient approximation (GGA)^43^ exchange correlation functional, an energy cutoff of 520 eV and a *k*-point mesh of at least 1000/atom. For the doped structures, an enumeration of all symmetrically distinct halide-vacancy orderings in a 2 × 2 × 2 supercell of Na_3_PS_4_ was carried out^44^, and the lowest energy configuration was used for all subsequent calculations and analyses, including *ab initio* molecular dynamics (AIMD) simulations. For phase diagram construction^45^, the pre-calculated energies of all phases in the Na-P-S-X systems (X = F, Cl, Br and I) other than those of primary interest in this work were obtained from the Materials Project database via the Materials Application Programming Interface^46^.

### Dopability analysis

To assess the likelihood of halide doping into Na_3_PS_4_, we estimated the neutral dopant formation energy using the formalism originally presented by Wei and co-workers^30^,^47^, 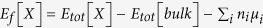, where *E*_tot_[X] and *E*_tot_[bulk] are the total energies of the structure with and without the neutral dopant X, respectively. *μ*_*i*_ is the atomic chemical potential of specie *i* that varies based on different experimental conditions; *n*_*i*_ indicates the number of atoms of specie *i* being added (*n*_*i*_ > 0) or removed (*n*_*i*_ < 0) from the pristine structure. In this work, the lower bound of the dopant formation energy was calculated, which is equal to the difference of decomposition energies between the doped and host materials^30^.

### Electrochemical analyses

The electrochemical stability of the solid electrolytes in contact with the electrodes was estimated using the grand potential approach proposed by Ong *et al*.^45^ which assumes that Na is the main mobile species. Under such conditions, the solid electrolyte-electrode interface is modeled as an open system with respect to Na. The relevant thermodynamic potential is then the grand potential, given as 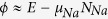, where *E* is approximated as the DFT total energy. It should be noted that the chemical potential of Na is related to the voltage vs Na/Na^+^ (*V*) via the following relation, with 
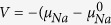
) where 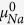 is the reference chemical potential of bulk Na metal.

### Ionic conductivity calculations

The diffusivity and conductivity of the Cl-doped t-Na_3_PS_4_ structure were calculated using AIMD simulations. Non-spin-polarized AIMD simulations were conducted in the *NVT* ensemble at 800–1400 K with a Nose-Hoover thermostat^48^,^49^. A smaller plane-wave energy cutoff of 280 eV and a minimal *Γ*-centered 1 × 1 × 1 *k*-point mesh were adopted. The time step of the simulations was 2 fs. The initial structure was fully relaxed at 0 K, and the volume was fixed for AIMD at elevated temperatures until the diffusivity was converged. No framework melting was observed in all simulations. All calculations were automated using an automated in-house AIMD workflow software^50^,^51^. The Na^+^ diffusivity (*D*) and conductivity (*σ*) can be extracted from the AIMD simulations using the following expressions: 
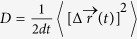
 where *d* is the dimensionality of diffusion ( = 3 for t-Na_3_PS_4_ structures); 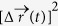 is the average mean square displacement (MSD) over a time duration *t*. The diffusivity was obtained by performing a linear fitting of the MSD vs. 2*dt*. Arrhenius plots were constructed to determine the activation energies and obtain extrapolated room-temperature diffusivities *D*_300K_. The room temperature Na ion conductivity *σ*_300K_ can then be derived from the Nernst-Einstein equation, 
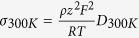
 where *ρ* is the molar density of diffusing Na ions in the unit cell; *z* = 1 is the charge of Na ions; and *F* and *R* are the Faraday’s constant and the gas constant respectively. *T* = 300 K was used in the above equation.

### Synthesis

Pure t-Na_3_PS_4_ was synthesized from reagent-grade Na_2_S (Alfa Aesar, 99%) and P_2_S_5_ (Sigma Aldrich, 99%). The precursors were ground in agate mortar and pestle in a molar ratio of 75:25, respectively. To introduce the chloride dopant, NaCl (Alfa Aesar, 99.99%) was mixed into the previous precursors following the chemical reaction (1.5 − *x*)Na_2_S + 0.5P_2_S_5_ + *x*NaCl → Na_3−*x*_PS_4−*x*_Cl_*x*_. The resulting mixtures were then sealed under vacuum in a quartz tube, heated to 1073 K (800 °C) for 4 hours, and then quenched in ice water. Subsequently, the sample was ground in a mortar and pestle and sealed in an ampoule to be heat treated at 693 K (420 °C) for 2 hours to stabilize the tetragonal phase. The samples were ground back into a powder with mortar and pestle, and re-pelletized. These pellets were then processed via spark plasma sintering (SPS). To prepare the sample, a 10 mm tungsten-carbide circular die was lined with graphite foil and the powder was placed in between two tungsten-carbide plungers, also coated with graphite. The entire setup was placed in the SPS chamber, and the sample was pressed to 100 MPa (100 MPa min^−1^), heated to 573 K (100 K min^−1^), and then allowed to dwell under these processing conditions for 5 minutes to reach a densified state. All synthesis steps were performed in a dry, inert (Ar) environment, unless otherwise stated.

### Characterization of solid electrolytes

The structural characterization was performed via X-ray diffraction (XRD). The data was collected by a Rigaku diffractometer over a 2*Θ* range of 30–60°, with a step size of 0.02° and a dwell time of 2 seconds. The beam was generated by a Cu-Kα source (40 kV, 100 mA). The sample was sealed under Kapton tape to prevent degradation or side-reactions during measurements.

Cross-sectional images of the SPS pellets were obtained using a Phillips XL30 scanning electron microscope (SEM). Pristine and doped pellets were suspended in an acrylic matrix that was polished for imaging. Iridium was sputtered onto the surface of the sample using an Emitech sputter chamber operating at 85 mA for 7 seconds. The sample was imaged using a 10 kV beam. Additionally, an elemental mapping analysis was conducted using an energy dispersive X-ray spectroscopy (EDX) aperture in the SEM. SEM and EDX were conducted under high vacuum.

### Electrochemical characterization

The electrochemical performance was evaluated via electrochemical impedance spectroscopy (EIS). The data was collected using a Solartron 1260 impedance analyzer operating from 1 MHz to 1 Hz with a DC bias of 0 V and an applied AC voltage of 25 mV. Carbon was used as blocking electrodes. The pellet was held between two titanium plungers serving as current collectors. The temperature dependence of conductivity was obtained by placing the cell in an electric furnace. The cell was ramped to 453 K (180 °C) from room temperature in 25 K increments. Prior to each measurement, the cell was held at the temperature for one hour to allow the system to reach thermal equilibrium. The sodium ion-migration activation energy was calculated from the slope of the Arrhenius plot. All measurements were taken in a dry, inert (Ar) atmosphere.

A full cell was assembled using a TiS_2_ composite cathode against a Na metal anode. TiS_2_ was mixed with t-Na_3−*x*_PS_4−*x*_Cl_*x*_, *x* = 6.25% in a 1:2 weight ratio. The doped (*x* = 6.25%) solid electrolyte (200 mg) was cold-pressed at 360 MPa in a 13 mm polyetheretherketone (PEEK) die. The cathode blende (10 mg) was cold-pressed at 360 MPa on top of the electrolyte layer. Pure Na metal was attached to a titanium current collector and cold-pressed into the die at approximately 30 MPa. The cell was cycled using Arbin battery cycler at room temperature. Cycling was performed at a C/10 rate for 10 cycles over a voltage window from 1.2 V to 2.4 V. The cell was cycled inside an Ar glovebox.

## Additional Information

**How to cite this article**: Chu, I.-H. *et al*. Room-Temperature All-solid-state Rechargeable Sodium-ion Batteries with a Cl-doped Na_3_PS_4_ Superionic Conductor. *Sci. Rep*. **6**, 33733; doi: 10.1038/srep33733 (2016).

## Supplementary Material

Supplementary Information

## Figures and Tables

**Table 1:** Formation energies of anion dopants in t-Na_3_PS_4_.

Dopant	*R*_X_/*R*_S_	*x* (%)	*E*_*f*_ (eV/dopant)
F	0.70	6.25	0.76
Cl	0.98	6.25	0.96
Br	1.07	6.25	1.11
I	1.21	6.25	0.99

Dopant formation energies *E*_f_ and ratio of halide to sulfide ionic radii (*R*_X_/*R*_S_) of the t-Na_3−*x*_PS_4−*x*_X_*x*_ (X = F, Cl, Br and I) at *x* = 6.25%.

**Table 2:** Room temperature ionic conductivity and activation energy of t-Na_3−*x*_PS_4−*x*_Cl_*x*_.

*x* (%)	AIMD simulations		Experiment	
	*σ*_300K_ (mS cm^−1^)	*E*_*a*_ (meV)	*σ*_303K_ (mS cm^−1^)	*E*_*a*_ (meV)
0	N/A	N/A	0.05	317
6.25	1.38 [1.04, 1.82]	232	1.14	249

Calculated AIMD and experimental Na^+^ conductivity and activation energy of the t-Na_3−*x*_PS_4−*x*_Cl_*x*_ superionic conductor. Values in the square brackets indicate the error range of the calculated ionic conductivity.

**Table 3:** Rietveld refinement of t-Na_3−*x*_PS_4−*x*_Cl_*x*_.

Pristine (*x* = 0%), Space Group 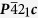					
*a* = *b* = 6.956 (5) Å, *c* = 7.088 (6) Å, *V* = 342.9 (5) Å^3^					
*R*_*b*_ = 3.86%, *R*_*wp*_ = 4.97%					
	*x*	*y*	*z*	Occ.	*B*_*iso*_ (Å^2^)
**Na1 (4*****d***)	0	0.5	0.426 (4)	2.00	2.54 (9)
**Na2 (2*****a***)	0	0	0	1.00	3.2 (1)
**P (2*****b***)	0	0	0.5	1.00	0.5 (6)
**S (8*****e***)	0.315 (3)	0.345 (2)	0.167 (2)	4.00	1.1 (6)
**Doped (*****x***** = 6.25%), Space Group** 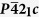					
*a* = *b* = 6.970 (4) Å, *c* = 7.092 (5) Å, *V* = 344.5 (4) Å^3^					
*R*_*b*_ = 4.29%, *R*_*wp*_ = 5.31%					
	***x***	***y***	***z***	**Occ.**	***B***_***iso***_ **(Å**^**2**^)
**Na1 (4*****d***)	0	0.5	0.428 (6)	1.99 (4)	3.0 (2)
**Na2 (2*****a***)	0	0	0	0.99 (4)	3.4 (3)
**P (2*****b***)	0	0	0.5	1.00	0.1 (1)
**S (8*****e***)	0.316 (4)	0.344 (3)	0.165 (3)	3.94 (4)	1.19 (9)
**Cl (8*****e***)	0.316 (4)	0.344 (3)	0.165 (3)	0.02 (4)	1.19 (9)

Rietveld refinement results of t-Na_3−*x*_PS_4−*x*_Cl_*x*_ systems, where *a*, *b* and *c* are lattice constants, and *V*, Occ., and *B*_iso_ are normalized cell volume, site occupation numbers, and isotropic atomic displacement parameters, respectively. Residual factors, R_b_ and R_wp_, for the pristine (*x* = 0%) composition are 3.86% and 4.97%, respectively; and for the doped (*x* = 6.25%) are 4.29% and 5.31%. For the pristine t-Na_3_PS_4_, the DFT calculated cell parameters are *a* = *b* = 6.99 Å, *c* = 7.12 Å, *V* = 348 Å^3^, in excellent agreement with the refinement results as well as those by Jansen *et al*. (ref. 27): *a* = *b* = 6.952 Å, *c* = 7.076 Å, *V* = 341.97 Å^3^.
