# Supplementary material for: Room-Temperature All-solid-state Rechargeable Sodium-ion Batteries with a Cl-doped Na3PS4 Superionic Conductor
Source: Sci Rep. 2016 Sep 20;6:33733. doi: 10.1038/srep33733 (PMC5028709; doi:10.1038/srep33733)
Supplement: Supplementary Information [file srep33733-s1.pdf]

Supplementary Information: Room-Temperature All-solid-state Rechargeable Sodium-ion Batteries with a Cl-doped Na_3PS_4 Superionic Conductor

Iek-Heng Chu[a], Christopher Kompella[a], Han Nguyen[a], Zhuoying Zhu, Sunny Hy, Zhi Deng, Ying Shirley Meng*, and Shyue Ping Ong*

Department of NanoEngineering, University of California, San Diego, 9500 Gilman Drive, Mail Code 0448, La Jolla, CA 92093, USA

a. These authors contributed equally to this work.

E-mail: shmeng@ucsd.edu; ongsp@eng.ucsd.edu

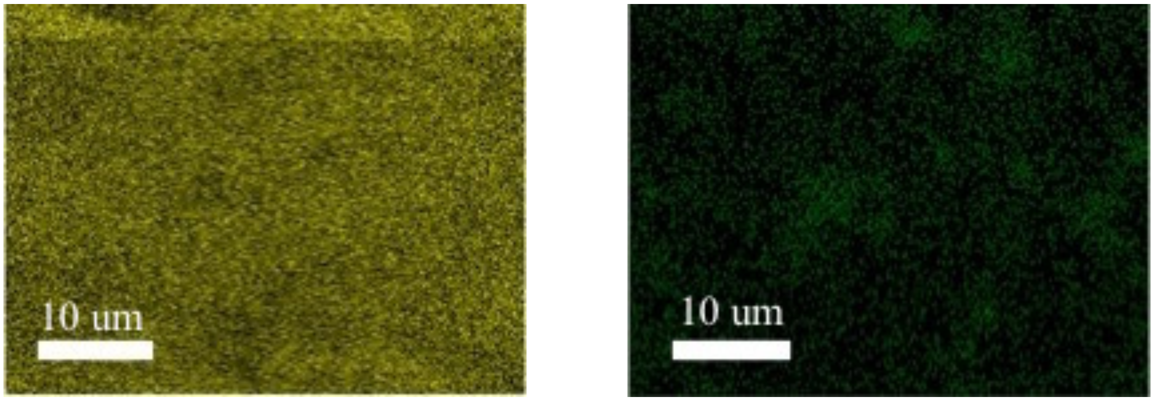

(a) **(b)**

Figure S1. EDX element maps of doped sample (*x* = 6.25%) for (a) S and (b) Cl. Maps correspond to SEM image (Figure 4e). Scale bar is 10 μm.

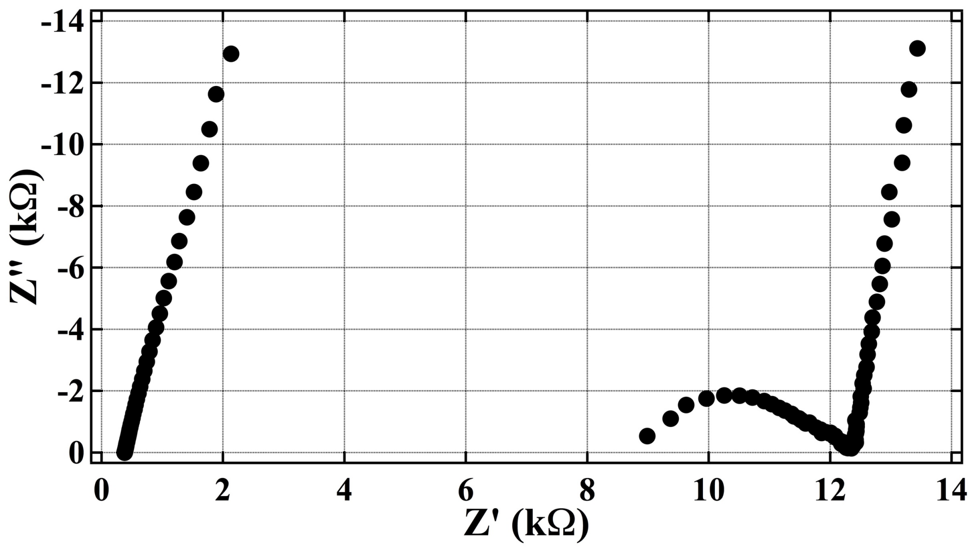

Figure S2. Room temperature (303 K) Nyquist plots of t-$Na_{3-x}PS_{4-x}Cl_x$. Pristine (x = 0%) performance shown right and doped (x = 6.25%) shown left.

Details of electrochemical impedance spectroscopy measurements

The ionic conductivity of the material was determined via electrochemical impedance spectroscopy (EIS). The total ionic conductivity has contributions from both the bulk and grain boundary, and was measured based on the intersection of the high-frequency capacitive semicircle and the low-frequency tail. The temperature-dependence of the ionic conductivity follows the equation $\sigma=\sigma_0 e^{(-Ea/kT)}$, so the slope of the Arrhenius plot can be used to calculate the Na^+ ion migration activation energy. The pristine (x=0%) pellet has the characteristic semicircle and spike typical of ionic conductors. On the other hand, the doped (x=6.25%) pellet does not have a resolvable semicircle though it was measured in the same frequency range as the pristine pellet. This phenomenon has been observed in several other materials, seemingly as the conductivity surpasses 1 mS cm^{-1}. The total room temperature impedances for the pristine and Cl-doped (x = 6.25%) pellets were roughly 12 kΩ and 400 Ω, respectively. By normalizing with the factor *(l/A),* we account for the thickness (*l*) and cross-sectional area (*A*) of the pellet, and the impedance values were then converted to ionic conductivity. The resulting ionic conductivities for the pristine and

Cl-doped (x = 6.25%) cases are 0.05 mS cm^{-1} and 1.14 mS cm^{-1}, respectively. In these EIS measurements, the pristine and Cl-doped (x = 6.25%) pellets were 2.49 mm thick with a diameter of 9.52 mm and 2.40 mm thick with a 9.67 mm diameter, respectively. It is worth pointing out that these dimensions are quite different from the solid-electrolyte layer in the full-cell assembly, which was 1 mm thick with a 12.95 mm diameter. Note also that the measured impedance of the Cl-doped pellet is of the same order of magnitude for the Si-doped Na_3PS_4 pellet in the previous work by Tanibata *et al.*, in which the resistance of their doped pellet is roughly 350 Ω.[1]

Table S1. Density measurements of t-$Na_{3-x}PS_{4-x}Cl_x$. The real density of the as-synthesized powders and the relative density of the spark plasma sintered pellets.

x	Real Density (g mL^{-1})	Relative Density (g mL^{-1})
0	2.268	2.085
0.0625	2.262	1.992

Details of sintering and density measurements

Though cold pressed sulfides, as compared to oxides, have been shown to be reasonably densified, there is still room for improvement. The increase in conductivity is, in part, owing to the densification of pellets via spark plasma sintering (SPS) which results in a reduction of grain boundary resistance. Grain boundary resistance can be separated into components characteristic of diffusion through the grain and those stemming from a reduced contact area due to porosity. While SPS does not address the intrinsic nature of the grain boundary, it does significantly reduce the porosity of the material, permitting greater contact area between grains. This explains why the ionic conductivity of the pristine t-Na_3PS_4 is an order of magnitude higher than previous reports.[2]

Density measurements were conducted to determine the real density of the as-synthesized powders and relative densities of the sintered pellets, and the results are shown in Table S1. The densities of the two materials are quite similar and we do not observe a significant difference between them. The real densities of powders were measured using a pycnometer purging helium. The real density measurement was carried out in an inert environment. The pristine and doped materials were 2.268 g mL^{-1} and 2.262 g mL^{-1}, respectively. These values are within 2% of the theoretical density and well within experimental error. The relative densities of the pristine and doped pellets were 1.99 g mL^{-1} and 2.08 g mL^{-1}, respectively, which is at least 90% of the theoretical density, 2.22 g mL^{-1}.[2] For this reason, SPS typifies a novel glass-ceramic processing technique which can be effective in both minimizing a material's grain boundary resistance and manufacturing viable all-solid-state batteries by attending to the issue of interfacial contact between the electrode and electrolyte.

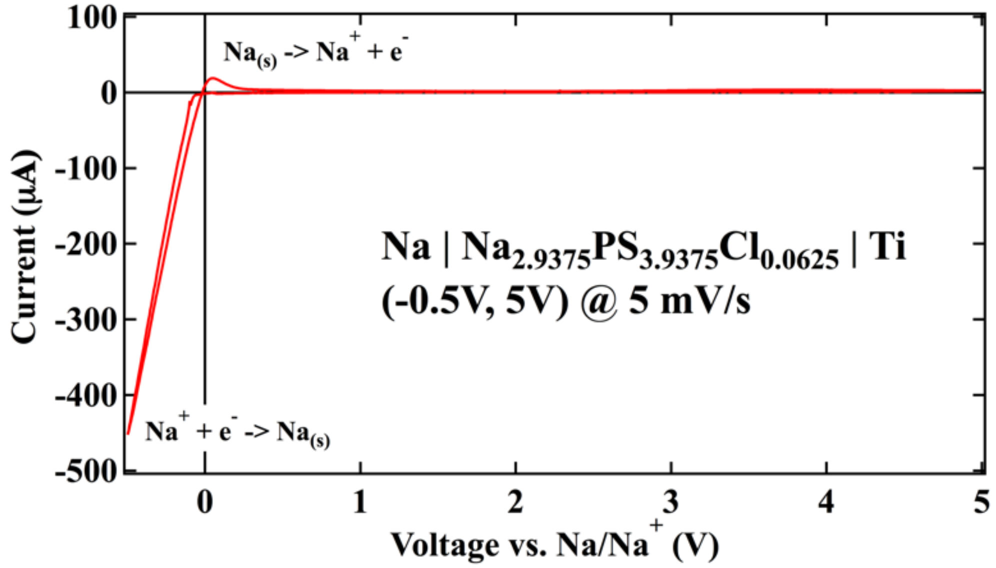
Na(s) -> Na+ + e-
Na | Na2.9375PS3.9375Cl0.0625 | Ti
(-0.5V, 5V) @ 5 mV/s
Na+ + e- -> Na(s)
Current (μA)
Voltage vs. Na/Na+ (V)

(a)

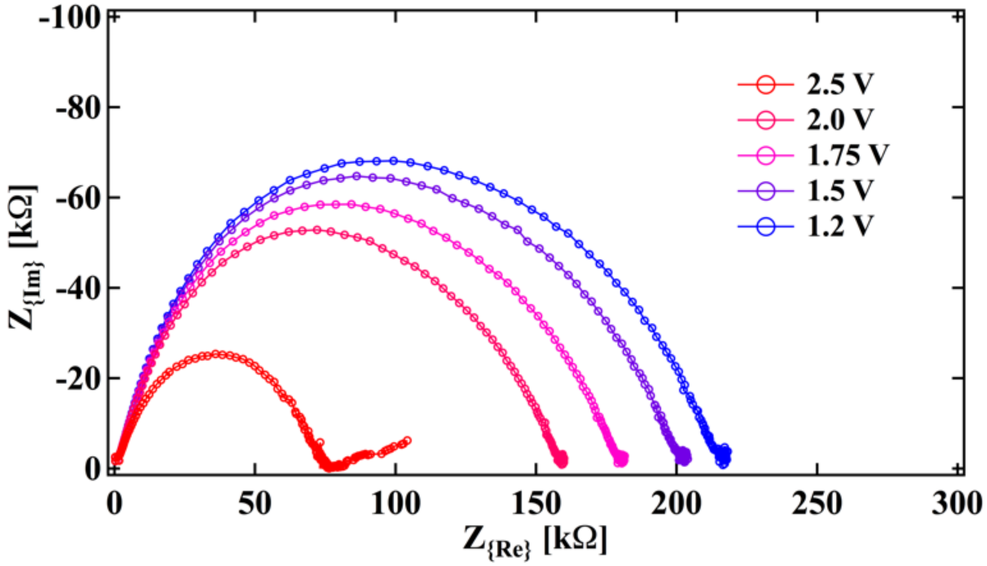
2.5 V
2.0 V
1.75 V
1.5 V
1.2 V
Z{Im} [kΩ]
Z{Re} [kΩ]

(b)

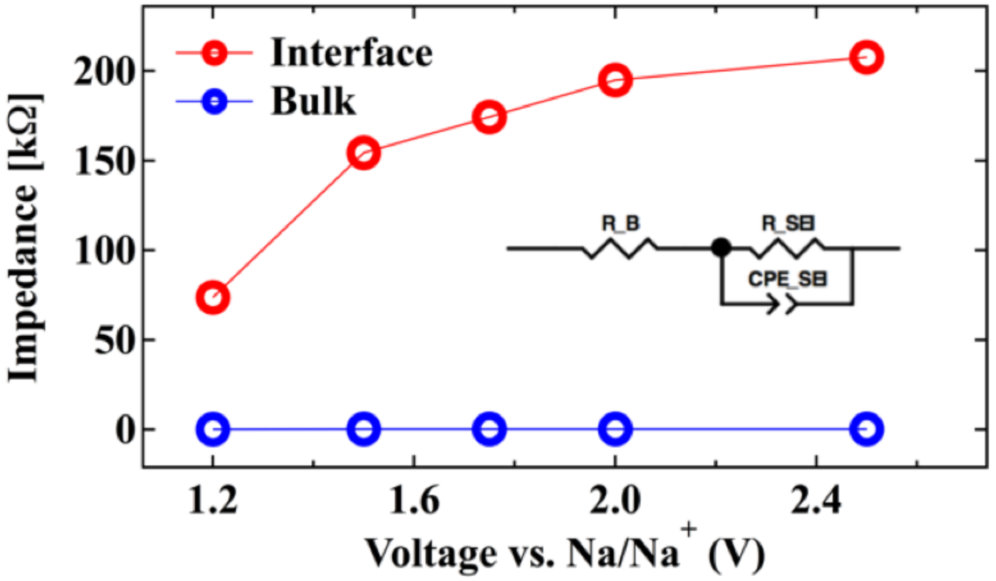

(c)

Figure S3. (a) Cyclic voltammogram of the t-$Na_{3-x}PS_{4-x}Cl_x$ / Na (x=0.0625) interface, swept from -0.5 V to 5 V. Here, a titanium plunger was used as the blocking electrode. The stripping and plating of Na metal is observed at 0 V vs. Na/Na^+, and no other anodic currents are noted up to 5 V. (b) Impedance plots of a discharging TiS_2 / t-$Na_{3-x}PS_{4-x}Cl_x$ / Na full-cell. Measurements were collected at 2.5 V, 2.0 V, 1.75 V, 1.5 V, and 1.2 V. (c) The impedance contributions of bulk and interface are deconvoluted using the results from the provided equivalent circuit.

Formation of stable solid-electrolyte interphase layers

The electrochemical stability of the Cl-doped t-Na_3PS_4 against Na metal was evaluated. Using a Solartron 1260/1287 analyzer, cyclic voltammetry was performed on a full-cell assembled as described in the Methods section, but using a titanium plunger as a blocking electrode instead of the TiS_2 cathode blende. The voltage was swept from -0.5 V to 5.0 V at 5 mV s^{-1}. The voltammogram shows cathodic and anodic peaks corresponding to the deposition and dissolution of sodium at 0 V vs. Na/Na^+ (see Figure S3a). No other currents are observed up to 5 V vs. Na/Na^+, suggesting a stable passivation layer at the anode-electrolyte interface.

Impedance measurements of the full-cell were also obtained during the first cycle discharge (see Figure S3b) using a Solartron 1260/1287. A cell assembled as described in the Methods section was discharged at a C/10 rate, but held at 2.5 V, 2.0 V, 1.75 V, 1.5 V, and 1.2 V while EIS measurements were performed. A 100 mV AC potential was applied and the frequency was swept from 1 MHz to 10 mHz. The cell was fitted using an equivalent circuit proposed by Oh and co-workers.,[3] including contributions from the bulk and interfacial phenomena.

Our results suggest the formation of solid-electrolyte interphase layers as the internal resistance of the cell increases dramatically over the course of the first discharge. We observe that the contributions from the bulk and grain boundary show hardly any deviation from their initial values, while a sharp increase is observed for the interfacial impedance. The capacitances from the interfacial contributions are 2 x 10^{-7} F, which is within the characteristic range of 10^{-7}-10^{-5} F.[4] Our results indicate that the increase in interfacial resistance is indeed attributable to a change in the nature of the interface.

Table S2. Dopant formation energies E_f and ratio of halide to sulfide ionic radii (R_X/R_S) of the cubic $Na_{3-x}PS_{4-x}X_x$ (X=F, Cl, Br and I) at x = 6.25%.

Dopant	R_X/R_S	x (%)	E_f (eV/dopant)
F	0.70	6.25	0.76
Cl	0.98	6.25	1.14
Br	1.07	6.25	1.27
I	1.21	6.25	1.09

Average voltage of Na_xTiS_2 ($0 < x < 1$)

For the TiS_2 charged cathode, we also estimated the average voltage for one electron per transition metal redox reaction. Using Perdew-Burke-Ernzerhof (PBE) generalized-gradient approximation (GGA) functional,[5] the voltage is around 1.58 V, in good agreement with the previous study.[6] On the other hand, we also computed the voltage using the Heyd−Scuseria−Ernzerhof (HSE) hybrid functional,[7,8] from which the calculated voltages are found to agree better with experiment.[9] The calculated HSE voltage for $NaTiS_2$ is 1.7 V, which is about 0.1 V higher than the PBE value.

References

1. Tanibata, N., Noi, K., Hayashi, A. & Tatsumisago, M. Preparation and characterization of highly sodium ion conducting Na3PS4–Na4SiS4 solid electrolytes. *RSC Adv.* **4,** 17120 (2014).
2. Jansen, M. & Henseler, U. Synthesis, structure determination, and ionic conductivity of sodium tetrathiophosphate. *J. Solid State Chem.* **99,** 110–119 (1992).
3. Oh, G., Hirayama, M., Kwon, O., Suzuki, K. & Kanno, R. Bulk-Type All Solid-State Batteries with 5 V Class LiNi 0.5 Mn 1.5 O 4 Cathode and Li 10 GeP 2 S 12 Solid

Electrolyte. *Chem. Mater.* **28,** 2634–2640 (2016).

4. Irvine, J. T. S., Sinclair, D. C. & West, A. R. Electroceramics: characterization by impedance spectroscopy. *Adv. Mater.* **2,** 132–138 (1990).

5. Perdew, J. P., Burke, K. & Ernzerhof, M. Generalized Gradient Approximation Made Simple. *Phys. Rev. Lett.* **77,** 3865–3868 (1996).

6. Ong, S. P. *et al.* Voltage, stability and diffusion barrier differences between sodium-ion and lithium-ion intercalation materials. *Energy Environ. Sci.* **4,** 3680 (2011).

7. Heyd, J., Scuseria, G. E. & Ernzerhof, M. Erratum: 'Hybrid functionals based on a screened Coulomb potential' [J. Chem. Phys. 118, 8207 (2003)]. *J. Chem. Phys.* **124,** 219906 (2006).

8. Heyd, J., Scuseria, G. E. & Ernzerhof, M. Hybrid functionals based on a screened Coulomb potential. *J. Chem. Phys.* **118,** 8207 (2003).

9. Lin, Y. *et al.* Thermodynamics, Kinetics and Structural Evolution of ε-LiVOPO4 over Multiple Lithium Intercalation. *Chem. Mater.* **28,** 1794–1805 (2016).
